# LoCoH: Nonparameteric Kernel Methods for Constructing Home Ranges and Utilization Distributions

**DOI:** 10.1371/journal.pone.0000207

**Published:** 2007-02-14

**Authors:** Wayne M. Getz, Scott Fortmann-Roe, Paul C. Cross, Andrew J. Lyons, Sadie J. Ryan, Christopher C. Wilmers

**Affiliations:** 1 Department of Environmental Science, Policy and Management, University of California, Berkeley, California, United States of America; 2 Mammal Research Institute, Department of Zoology and Entomology, University of Pretoria, Pretoria, South Africa; 3 Northern Rocky Mountain Science Center, U.S. Geological Survey, Montana State University, Bozeman, Montana, United States of America; 4 Department of Ecology, Montana State University, Bozeman, Montana, United States of America; 5 Environmental Studies Department, University of California, Santa Cruz, California, United States of America; Imperial College, United Kingdom

## Abstract

Parametric kernel methods currently dominate the literature regarding the construction of animal home ranges (HRs) and utilization distributions (UDs). These methods frequently fail to capture the kinds of hard boundaries common to many natural systems. Recently a local convex hull (LoCoH) nonparametric kernel method, which generalizes the minimum convex polygon (MCP) method, was shown to be more appropriate than parametric kernel methods for constructing HRs and UDs, because of its ability to identify hard boundaries (e.g., rivers, cliff edges) and convergence to the true distribution as sample size increases. Here we extend the LoCoH in two ways: “fixed sphere-of-influence,” or *r*-LoCoH (kernels constructed from all points within a fixed radius *r* of each reference point), and an “adaptive sphere-of-influence,” or *a*-LoCoH (kernels constructed from all points within a radius *a* such that the distances of all points within the radius to the reference point sum to a value less than or equal to *a*), and compare them to the original “fixed-number-of-points,” or *k*-LoCoH (all kernels constructed from *k*-1 nearest neighbors of root points). We also compare these nonparametric LoCoH to parametric kernel methods using manufactured data and data collected from GPS collars on African buffalo in the Kruger National Park, South Africa. Our results demonstrate that LoCoH methods are superior to parametric kernel methods in estimating areas used by animals, excluding unused areas (holes) and, generally, in constructing UDs and HRs arising from the movement of animals influenced by hard boundaries and irregular structures (e.g., rocky outcrops). We also demonstrate that *a*-LoCoH is generally superior to *k*- and *r*-LoCoH (with software for all three methods available at http://locoh.cnr.berkeley.edu).

## Introduction

Ecology is currently undergoing a revolution in terms of our ability to collect large sets of data with unprecedented precision on the position of individuals in the landscape (e.g. plus-minus several meters using current GPS technology [1]) at regularly spaced intervals of time. This revolution is leading to the emergence of movement ecology, a new subfield of ecology [2]. GPS position data is often used to construct home ranges (HRs) [3]–[6] or utilization distributions (UDs) [7]–[13], where the former are bounded areas used by animals for some defined purpose (e.g. foraging or seeking mates), while the latter are represented by isopleths demarcating regions in space with different probabilities or rates of usage by individuals.

Currently, the boundary of the HR is commonly delimited using the 95% isopleth of an unbounded UD, where the UD is typically constructed using the symmetric bivariate Gaussian (i.e. a parametric) kernel method [14]–[19], although other methods may be preferred when the UD is multimodal [20]. For comparative and other reasons enumerated below, bounds on the innermost 95% of the data are also used to estimate the areas of HRs even for methods of construction that are able to produce HRs bounded by a 100% isopleth of a UD (e.g. the minimum convex polygon—MCP, bounded parametric kernel methods, our LoCoH methods). In the future, use of the 95% isopleth to bound HRs may change in view of Börger et al.'s [21] recent study in which they recommend estimating the area of HRs using isopleths in the 50–90% range. They demonstrate that using isopleths in this range produces area estimates that are less biased by sample size than when using isopleths above 90% or below 50% (the latter sometimes being used to estimate core areas of HR use).

The reasons for omitting outlying points in estimating the size of HRs are threefold: (1) locations based on relatively inaccurate triangulation of radio collars result in imprecise location estimates (this is philosophically consistent with the parametric kernel methods, such as the radially symmetric—i.e. one parameter—bivariate Gaussian or harmonic kernels, that associate a smooth distribution with each data point); (2) HR area estimates using MCP and parametric kernel construction methods are very sensitive to outlying points [21]; and (3) outlying points may well reflect exploratory animal movements rather than those necessary for survival and reproduction. The first of these three points is no longer relevant for methods applied to GPS data since these data are spatially precise [22].

Here we describe extensions to a recently developed local convex hull (LoCoH) approach [23] that produces bounded HRs and has been shown to have superior convergence properties compared to the parametric kernel methods used in constructing HRs and UDs. This LoCoH method is both a generalization of the minimum convex polygon (MCP) method and essentially a non-parametric kernel method. LoCoH applies the MCP construction to a subset of data localized in space, and the local convex polygon (i.e. local hull) is constructed using the *k*-1 nearest neighbors of each data point, thereby producing a set of nonparametric kernels whose union is the UD. Thus LoCoH uses kernels with forms arising directly out of the data, unlike parametric kernels that have a form specified in most cases by a one parameter function (e.g. symmetric bivariate Gaussian centered on the data point with width parameter *h*), even though the union of these parametric kernels can produce rather irregular surfaces with multiple peaks.

The advantage of LoCoH's direct use of data becomes evident when constructing UDs from data influenced by idiosyncratic geometries such as geomorphological boundaries and holes (e.g. lakes or rocky outcrops) associated with the space over which animals move [23]. In particular, as illustrated in examples considered here and elsewhere [23], [24], LoCoH methods are more adept than parametric kernel methods at locating such geographical features as reserve boundaries, rivers, lakes, inhospitable terrain, and so on. Further, these features can be assessed automatically by linking LoCoH constructions with spectral images provided by new remote sensing technologies that have resolutions matching or exceeding those of the data (e.g. 1–10 m resolution SPOT imagery, Quickbird and Superbird images, IKONOS satellite imagery [22]). Statistical analyses can then be carried out to address ecological questions relating, among other things, to resource use [25] or social behavior [26].

In this paper, we present two modifications of the “fixed *k*” LoCoH method, which has been referred to as the *k*-NNCH (k-nearest neighbor convex hulls) because each local kernel is a *k*-point convex hull constructed from a root point and its *k*-1 nearest neighbors [23]. The first modification is a “fixed radius” *r*, or *r*-LoCoH, method in which all the points in a fixed “sphere of influence” of radius *r* around each root point are used to construct the local hulls. The second modification is an adaptive, or *a*-LoCoH, method in which all points within a variable sphere around a root point are used to construct the local hulls such that the sum of the distances between nearby points and the root point is less than or equal to *a*. Thus the adaptive method allows the number of points involved in the construction of the LoCoH kernels to increase with increasing density of data.

After presenting a description of the methods and reviewing the MSHC approach (minimum spurious hole covering—see [23]) to selecting an appropriate value for *k*, *r*, or *a*, we compare the performance of parametric kernel and LoCoH methods in estimating UD isopleths from data generated from known distributions with challenging spatial features (e.g. narrow valleys or corridors). We then compare results obtained from the application of parametric and LoCoH kernel methods to both manufactured and real data, the latter from GPS collars placed on African buffalo in the Kruger National Park, South Africa. In particular, we demonstrate the superior performance of LoCoH compared with parametric kernel methods in the context of estimating the size of HRs and delineating geological and ecological features in home ranges.

Finally, we note that links to software for the implementation of LoCoH using ArcView/ArcGIS, or in the R Statistical package Adehabitat, or as a web application can be found at http://locoh.cnr.berkeley.edu.

## Methods

### A. Constructions

#### Fixed number of points: k-LoCoH

As elaborated in more detail in Getz and Wilmers [23], the method begins by constructing the convex hull associated with each point (the root) and its *k*-1 nearest neighbors. The union of all these hulls is finite and can be used to represent the home range of the associated individual. (For a method based on α-hulls see Burgman and Fox [27]. To obtain a UD, the hulls are ordered from the smallest to the largest, where the smallest hulls are indicative of frequently used areas. By progressively taking the union of these from the smallest upwards, until *x*% of points are included (with some rounding error), the boundaries of the resulting union represents the *x*% isopleth of the densest set of points in the UD. Depending on convention the HR can be defined as the area bounded by the 100% isopleth of the UD or, for purposes of comparison, the 95% isopleth which is the one most commonly used for UDs constructed from more traditional, particularly non-compact, kernels such as the symmetric bivariate Gaussian.

#### Fixed radius: r-LoCoH

Instead of choosing, as in the fixed *k* LoCoH, the *k*-1 nearest neighbors to each point, we use all points at distance *r* or closer to the root point to construct the local hull associated with the root and all points within a “sphere of influence” of radius *r*. Since all the local convex hulls now are approximately the same size, to construct the UD we sort these hulls from those containing the most points to those containing the fewest, with a size (area) sorting only being used to order hulls containing the same number of points. As before, we progressively take the union of hulls from most to fewest points and smallest to largest when they have the same number of points until *x*% of points (with some rounding error) are included. Also, as before, the boundaries of the resulting union represent the x% isopleth of the densest set of points in the HR.

If *r* is sufficiently small so that some points have only one or no neighbors then in the one-neighbor case the point is connected to the construction by a line, while in the no-neighbors case the point is isolated from the construction. In both cases, the points do not contribute any area to the construction. If the proportion of such points is *p* then the area bounded by the construction is the 100(1−*p*)% isopleth. If construction of a 100% isopleth is needed, then the algorithm can be modified to include at least the two nearest neighbors irrespective of the value of *r*.

The above method for constructing a fixed radius LoCoH is reminiscent of fixed kernel methods that use kernels with finite support, such as the uniform or Epanechnikov kernels [16], except in LoCoH the elements are data dependent and hence variable in shape while the parametric kernels have the same repeated element associated with each point.

#### Adaptive or a-LoCoH method

The adaptive or *a*-LoCoH method uses all points within a variable sphere around a root point such that the sum of the distances between these points and the root point is less than or equal to *a*. Essentially, this method adjusts the radius of the circle that circumscribes each local convex hull, such that smaller convex hulls arise in high use areas, thereby providing more clearly defined isopleths in regions where data are more abundant. Thus, for example, the *a*-LoCoH method is particularly useful in defining UD boundaries that arise when an individual regularly visits the shore of a lake, the edge of a cliff, or the bank of a river. Also, provided the value *a* exceeds the sum of the two greatest distances between points in our data set, the construction will always produce the 100% isopleth while keeping the radius of LoCoH elements small in high density regions of the data. On the other hand, if *a* does not exceed the sum of the two greatest distances between points in our data set, then to obtain the 100% isopleth we need to specify that at least the two nearest neighbors are always included irrespective of the value of *a*.

#### Rules for selecting k, r or a

**Figure 1:**
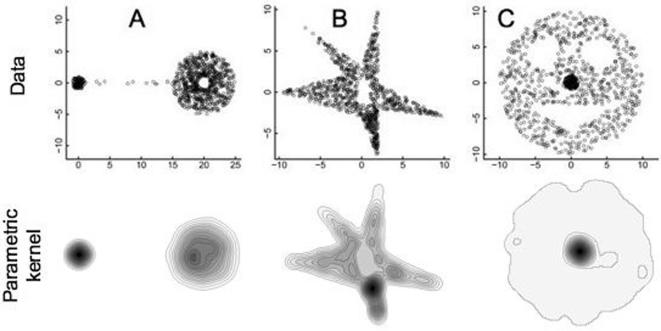
The actual points used in the analysis, selected at random within boundaries defined in the methods to conform with the specified isopleth rules, are plotted here in the upper row for data sets A, B, and C. For each set, the 20% isopleth surrounds the densest aggregation of points that appear as relatively black areas in each of the plots. UDs constructed using the fixed kernel least-squares cross-validation method for these data are illustrations in the lower row (sizes have been adjusted to provide visual correspondence—where precise estimates of the fits are given in Table 1).

**Figure 2:**
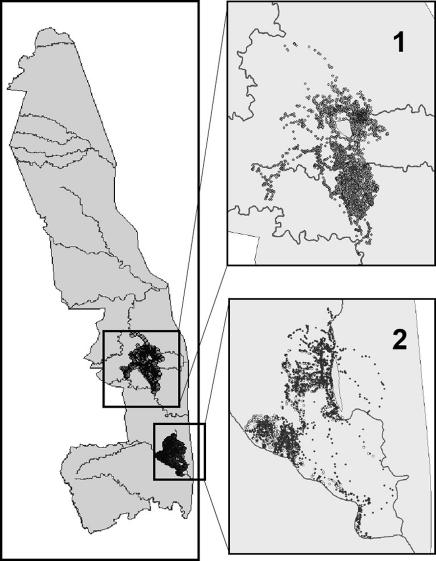
Kruger National Park, showing the location of the four collared buffalo used in the empirical data test of the study. The Satara and Lower Sabie regions are shown as insets 1 and 2, respectively.

For relatively low values of *k*, *r*, or *a*, the resulting LoCoH construction from the union of the LoCoH elements associated with each data point may contain many unused areas (or holes) that disappear with increasing *k*, *r*, or *a*. For HRs with known topologies (i.e. where the number of holes that the UD should contain is known ahead of time) the “minimum spurious hole covering” (MSHC) rule [23] may be used to select the smallest value of *k*, *r*, or *a* that produces a covering that has the same topology as the given set (e.g. see Figs. 1 and 2). If the topology of the UD is not known, we can guess its genus (number of holes) by identifying relatively large physical features, such as lakes, mountain peaks, or inhospitable habitats. We expect these objects to produce real holes in the data that should be reflected in the UD construction. Of course, real holes at scales that are relatively small compared with the size of the home range may be missed. Differences between real and spurious holes in LoCoH constructions may be evident in plots of area covered by the UD against the value of the parameter *k*, *r*, or *a*: with increasing parameter values the estimated area may level off once all spurious holes are covered [23]–[24], but should increase again when one or more real holes becomes totally or partially spuriously covered. Identifying these plateaus in UD construction determines the value to use. We denote these values by *k̂*, *r̂* and *â*. Only experience with the method, however, will reveal appropriate methods for deciding when this leveling off has been achieved. While this MSHC rule is subjective, we show in this paper that the *a*-LoCoH method is remarkably robust to changes in the parameter *a*.

**Table 1:** Total Error, with Type I and Type II Errors in parentheses for manufactured data sets **A**–**C**, as a percentage of total home range size, is listed for estimates obtained using the three LoCoH methods (100% isopleths and optimal—that is error minimizing—values k*, r* and a*) and the Gaussian kernel (GK) method (95%, 99% and optimal isopleths).

Data (true area)	A (85.3 units)	B (68.0 units)	C (257.0 units)
*k*-LoCoH	13.4% (8.8%, 4.6%)	**8.7%** (4.9%, 3.8%)	9.0% (6.7%, 2.3%)
*k* &	15	27	17
*r*-LoCoH	15.0% (8.4%, 6.6%)	10.3% (5.9%, 4.4%)	8.8% (5.6%, 3.2%)
*r* &	2.0	1.0	1.75
*a*-LoCoH	**8.8%** (5.9%, 2.9%)	**8.7%** $ (4.6%, 4.0%)	**8.6%** (5.4%, 3.2%)
*a* &	21.0	19	19
GK 95%	27.3% (22.2%, 5.2%)	30.3% (2.9%, 27.4%)	20.2% (14.9%, 5.2%)
GK 99%	20.9% (10.4%, 10.4%)	56.6% (0.4%, 56.2%)	15.0% (3.9%, 11.1%)
GK minimum*	20.9% (10.4%, 10.4%)	22.2% (10.9%, 11.5%)	14.6% (6.1%, 8.6%)
(isopleth)**	(99%)	(87.5%)	(98.25%)

The best estimate is in bold type.

& optimal values reflect integer resolution for *k* and 0.25 resolution for *r* and *a*; *minimizes total error; **^$^**0.1 difference in sum due to rounding; **search resolution is a quarter of a percent apart.

For our manufactured data sets where the boundaries of the areas are known, or in cases of field data where the boundaries of particular holes are known, values of the parameters for *k*, *r*, and *a* can be obtained by minimizing the sum of Type I and II errors (Type I errors arise from excluding regions that are part of the HR while Type II errors arise from including regions that are not: see [23]) in terms of how well our LoCoH methods identify the boundaries of the areas in question. As a starting point for finding these optimal values, denoted by k*, r*, and a*, a set of heuristic values, denoted by *k*_1_, *r*_1_, and *a*_1_ respectively, were selected using the following “rules of thumb:”

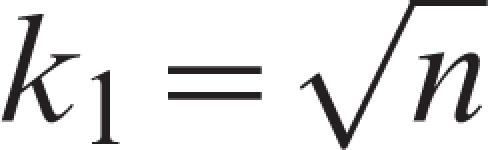
 values (*n* is the number of points in the set)
- *r*_1_ is half of the maximum nearest neighbor distance between points (i.e. the radius of a sphere that will allow all points to be joined to at least one additional point)
- *a*_1_ is maximum distance between any two points in the data set.

#### Parametric kernel constructions

For purposes of comparison we constructed UDs using symmetric bivariate Gaussian kernels. Although we sought to use the optimized value for the width parameter, *h*, using the least-squares cross-validation (LSCV) method (see [18]; but see [28] for problems with this method), for one of the generated data sets and for the buffalo data, the method did not converge using either the R-Adehabitat toolbox or the Animal Movement Extension for ArcView 3.x [29]. This is a common problem with the method so we used Silverman's ad-hoc method instead for generating the width parameter *h* [14].

### B. Data

#### Manufactured Data

We manufactured three datasets (Fig. 1 A–C) with known 20% and 100% isopleths so that we could use these to compare the accuracy of our three methods.

Dataset A: The 100% isopleth is constructed from a ring centered at the (*x,y*) = (20,0) with an inner radius of one and an outer radius of five. The ring contains 78% of the points and was connected by a corridor width of 14 and a height of 0.5 containing 2% of the points. This corridor connects to a circle located at (0, 0) with a radius of one that contains 20% of the highest density points in the construction. Thus this circle is also the 20% isopleth. We randomly distributed 1,000 points in the dataset according to the isopleth rules: 78% in the ring, 20% in the small dense circle and 2% in the connecting corridor. The area bounded by the densest 20% and the 100% isopleths is 3.1 and 85.3 units respectively.

Dataset B: The polygon defined by joining lines to the ordered set of points (−10, 0), (−2, 2), (−7, 8), (0, 3), (2, 10), (2, 1), (10, −3), (2, −2), (2, −8), and (0, −3) is the 100% isopleth boundary for these data. The 20% densest point aggregation is within the triangle (2, −2), (2, −8), and (0, −3). A rectangular hole in the data set is bounded by the lower left corner of (−0.5, −1.5), and has a width and height of 1.5 and 3 respectively. We randomly distributed 1,002 points in the dataset concordant with the isopleth rules, but otherwise at random. The area bounded by the densest 20% and the 100% isopleths is 6.0 and 68.0 units respectively.

Dataset C: The 100% isopleth was created from a circle centered at (0, 0) and radius 10, with two circular holes of radius 2.2 centered at (4, 4) and (−4, 4) and a triangular hole with vertices (−6.5, −3), (6.5, −3), and (0, −7). We constructed the 20% isopleth from a circle centered at (0, 0) with a radius of one. Lastly, we randomly distributed 1,002 points in the dataset concordant with the isopleth rules, but otherwise at random. The area bounded by the densest 20% and the 100% isopleths is 3.1 and 257.0 units respectively.

#### Buffalo data

We collected field data on African buffalo movements using VHF and GPS collars place on individuals from November 2000 to August 2006 in the Satara and Lower Sabie regions of the Kruger National Park. For the purposes of demonstrating the LoCoH methodology we restrict our analyses to GPS recordings of locations taken once an hour from four adult females over the following periods of times: female T13, July 15, 2005 to Oct 29, 2005; female T15, Sept 16, 2005 to Feb 16, 2006; female T7, Sept 15, 2005 to Jan 29, 2006; female T16, July 27, 2005 to October 8, 2005. These data were collected in decimal degrees and re-projected to Universal Transverse Mercator (UTM) [WGS84, Zone 36S] in ArcGIS 9. These data represent two buffalo at each of two sites in Kruger National Park: the first is the Satara region (T07 and T15) and the second is the Lower Sabie region (T13 and T16) (Fig. 2). In both regions, areas within the range of the buffalo are known to be physically inaccessible. A 7.7 km^2^ fenced exclosure exists in the Satara region while a small ridge (∼4.15 km^2^) that is too steep for the buffalo to climb exists within the Lower Sabie region. Both the exclosure and ridge serve as “known holes” that can be used to assess the performance of the methods, as discussed below.

### C. Analysis

#### Error analysis using manufactured data

For each of the datasets we constructed *k-*, *r-*, and *a-*LoCoH UDs over a range of parameter values. In every case, we calculated the Type I and Type II errors associated with the 20% and 100% isopleth constructions. We took the total error to be the sum of Type I and Type II errors for the isopleth in question; although for some applications, if the relative importance of Type I and II errors differs, a weighted sum can be used. Here we simply define the optimal *k*, *r*, or *a* to be the values that minimize the total error for the corresponding method. As discussed above, for the symmetric bivariate Gaussian kernel method we followed the convention of using the 95% isopleth to bound the UDs, but also included the 99% isopleth for purposes of comparison. We then identified the isopleth that minimized the total error.

We constructed images of the resulting LoCoH UDs for our optimal parameter values, as well as half and twice the optimal values.

Lastly, we examined how the total error of the UDs constructed using the different methods changed as we used different sample sizes. We generated random samples containing 1000, 800, 600, 400, and 200 points using the specifications and isopleth rules outlined earlier for each manufactured dataset. We repeated this process 15 times (this number is relatively low but suffices if we are generating estimates purely for comparative purposes among methods) as a way of generating error estimates (i.e. for a total of 75 samples per dataset). We located the optimal value of *k*, *r*, and *a* for each sample and plotted the resulting total error as a function of sample size.

#### Error analysis using Buffalo data

For purposes of comparison, we generated UDs for each of the four individuals using each of the 4 different methods. Since we were uncertain over what range of values we should explore the performance of our MSHC algorithm, we initially constructed UDs using our heuristic rules for selecting *k*_1_, *r*_1_, and *a*_1_. For the two data sets from Satara, for which the exclosure is precisely known, we then assessed to what extent the known holes were covered with these initial parameter guesses and used this information to locate the values of the parameters where the known holes were completely covered for the first time—that is the MSHC parameter values *k̂*, *r̂*, and *â*. For all three methods we always ensured that at least the two nearest neighbors were included: thus in all cases the 100% isopleth could be constructed. We then divided the intervals [0,*k̂*], [0,*r̂*], and [0,*â*] into 20 subsections and calculated the proportion of the known holes covered for each of the 20 parameter values in question with respect to the two data sets under consideration.

## Results

### Manufactured Data

**Figure 3:**
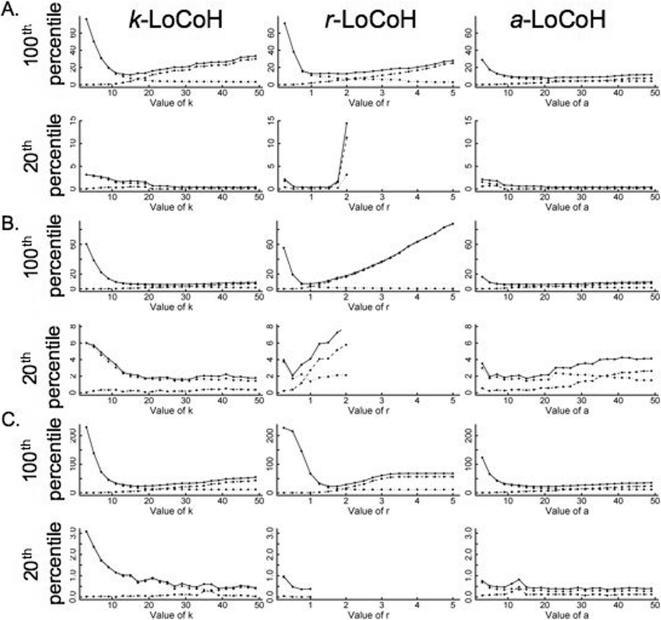
Type I (dotted line), Type II (dashed line) and Total Error (solid line) (percentages) associated with the construction of 100% and 20% isopleths are plotted for the *k*-LoCoH, *r*-LoCoH, and *a*-LoCoH methods as a function of the parameters, *k*, *r* and *a* respectively for the three data sets (A, B, and C).

For each of the three data sets we plot in Figs 3A–C the total errors associated with the *k*-LoCoH, *r*-LoCoH, and *a*-LoCoH constructions of home ranges (100% isopleth) and the 20% isopleths as a function of the parameters, *k*, *r* and *a* respectively. In the case of the home range constructions, the optimal value of *r* (i.e. the value that minimizes the total error associated with the *r*-LoCoH constructions) is evident from the graphs. For the *k*-LoCoH home range constructions, the optimum *k* is obvious for data set A, but less so for data sets B and C. On the other hand, the total error curves for the *a*-LoCoH home range construction become rather flat beyond small values of *a* and the optimum value is not that obvious from the graph (which is why, as we will see below, that this method is the most robust of the three LoCoH methods). For all the cases the value of the parameters that minimize total error for the HR are given in Table 1 where, for purposes of comparison, the errors associated with the symmetric bivariate Gaussian kernel construction are listed for the 95% isopleths, the 99% isopleths, as well as the isopleth constructions that minimized the total error (to within a resolution of isopleths differing by ¼%). All three LoCoH methods have errors that are considerably lower than those of the symmetric bivariate Gaussian kernel (GK) constructions. In particular, the *a*-LoCoH estimates were either best (data sets A and C) or tied for best (data set B) with error levels between 8.6–8.8%, while the optimal GK estimate error levels where 20.9%, 22.2%, and 14.6% for data sets A–C respectively: that is, error rates of around 2–3 times those of the *a*-LoCoH constructions.

Also note in Table 1 that the Type I and Type II errors associated with the three LoCoH methods are relatively similar, whereas this is not generally true for the symmetric bivariate Gaussian kernel method. In addition, the plots of error levels for the LoCoH constructions (Fig. 3) indicate that *a*-LoCoH constructions were less sensitive than the *k*- and *r*-LoCoH constructions to variation in the proportional changes to the values of the parameters around their optimal values. With regard to errors associated with estimating the construction of the 20% isopleth, the *r*-LoCoH method breaks down as soon as the value of *r* increase beyond a critical value (e.g. in datasets A and C around the radius of the core set of points in the data sets), while the *k*-LoCoH and *a*-LoCoH methods are more reliable, with the former actually performing better for data set B and the latter performing better for data sets A and C.

**Figure 4:**
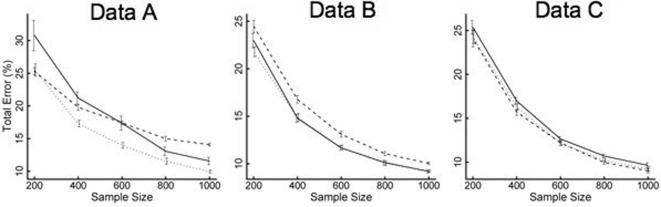
The effect of sample size on the optimal (i.e. error minimizing) value of parameters, *k*, *r* and *a* and total errors associated with the construction of the 100% isopleth using the *k*-LoCoH (solid line), *r*-LoCoH (dashed line), and *a*-LoCoH (dotted line) methods respectively for the three data sets (A, B, and C). Mean and standard error for fifteen randomly generated datasets for each sample size are plotted.

For each of the three data sets, the errors of the LoCoH models are plotted as a function of sample size for the optimal (i.e. error minimizing) values of the parameters (Figs. 4A–C). For all values and all cases the errors decrease with sample size. For dataset A, *r*-LoCoH moves quickly from performing the best (but well within the error bars) for the smallest sample size to performing by far the worst for the largest sample size. *a*-LoCoH is consistently strong throughout this dataset. For dataset B, *k-* and *a-*LoCoH have nearly identical accuracy except for the smallest sample size where *a*-LoCoH obtains a smaller error. *r*-LoCoH lags behind across all sample sizes in this dataset. In dataset C, the three methods perform roughly equally (within the error bars) with *r*-LoCoH appearing to be slightly superior, followed by *a*-LoCoH, and lastly by *k*-LoCoH.

The optimal value k* increased with sample size for all three data sets (Table 2) with the heuristic initial guess 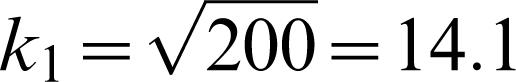 very close to the optimal value for all three sets of data when *n* = 200 but not as close when *n* = 1000: in the latter case a rule of 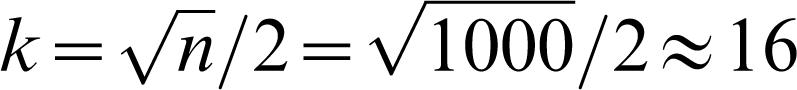 works better than the heuristic rule. As expected, the optimal value of *r* decreased with increasing point density. The optimal value of *a* also decreased, but not strictly monotonically (Table 2). The heuristic rule for *r* produced a value *r*_1 _that was generally lower than the optimal r* by factor of 1.5 to 3. On the other hand, the heuristic rule for *a* produced a value *a*_1 _that was surprisingly close to a*, in some cases being very close, and others being too high or low by a factor of only 0.2.

**Table 2:** Comparison of our heuristic rules for choosing initial parameter values *k*_1_, *r*_1_ and *a*_1_ and optimal parameter values k*, r* and a* for the manufactured data.

Data (true area)	*k*-LoCoH		*r*-LoCoH		*a*-LoCoH	
	*k*_1_	k*	*r*_1_	r*	*a*_1_	a*
**A:** 200 points	14.1	13.5 (0.70)	1.41 (0.18)	2.37 (0.05)	25.5 (0.04)	24.9 (1.00)
1000 points	31.6	14.5 (0.42)	0.56 (0.00)	1.67 (0.06)	25.7 (0.00)	23.3 (0.84)
**B:** 200 points	14.1	11.9 (0.48)	0.74 (0.08)	1.51 (0.04)	18.4 (0.12)	15.5 (0.74)
1000 points	31.6	20.6 (0.60)	0.57 (0.00)	0.79 (0.00)	19.6 (0.00)	14.0 (0.23)
**C:** 200 points	14.1	12.7 (0.42)	1.00 (0.03)	3.10 (0.05)	19.7 (0.03)	24.4 (0.82)
1000 points	31.6	17.3 (0.27)	0.46 (0.00)	0.79 (0.03)	19.9 (0.00)	20.7 (0.59)

Mean values are given with standard error in parentheses calculated over 15 different samplings of the data.

**Figure 5:**
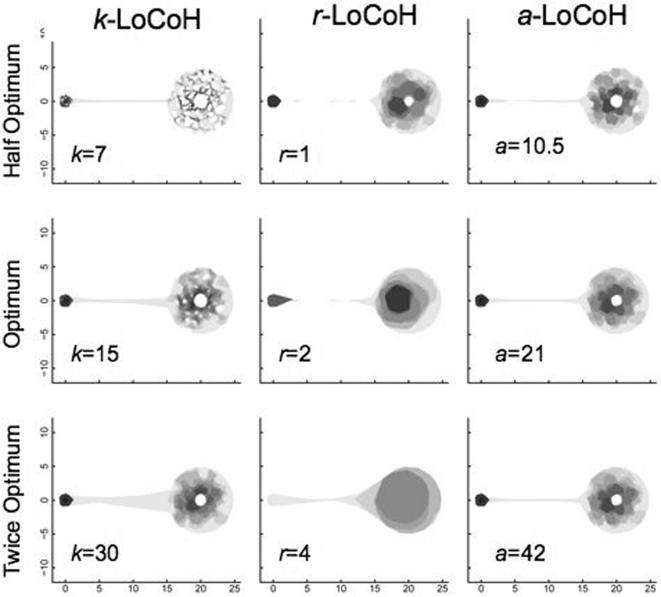
Illustrations of UDs constructed for data set A using *k*-LoCoH, *r*-LoCoH, and *a*-LoCoH methods with half, actual, and twice the optimal *k*, *r* and *a* parameter values. The darkest to lightest areas represent ascending decile areas from the 10^th^ to 100^th^ percentile isopleths.

In Figs. 5–7, the UDs for the half-optimal, optimal, and twice-optimal parameter values are plotted for data sets A, B and C, respectively. These constructions illustrate that the *a*-LoCoH method is the least sensitive to changes in the value of the parameter *a*, *r*-LoCoH the most sensitive, and *k*-LoCoH is intermediate. Moreover, of the three methods, *k*-LoCoH is most likely to create spurious holes (Type I errors) at half the optimal *k* value, while *r*-LoCoH is most likely to fill in real holes (Type II errors).

**Figure 6 pone-0000207-g006:**
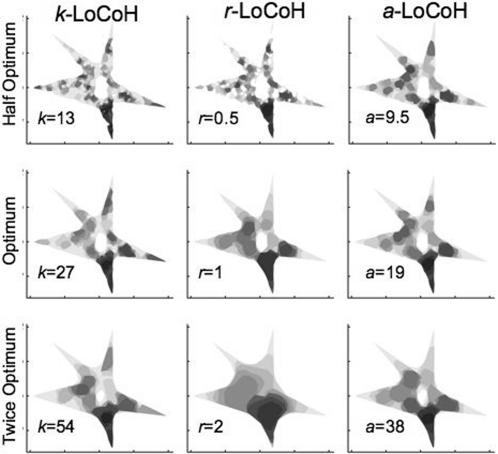
Illustrations of UDs constructed for data set B using *k*-LoCoH, *r*-LoCoH, and *a*-LoCoH methods with half, actual, and twice the optimal *k*, *r* and *a* parameter values. The darkest to lightest areas represent ascending decile areas from the 10^th^ to 100^th^ percentile isopleths.

**Figure 7:**
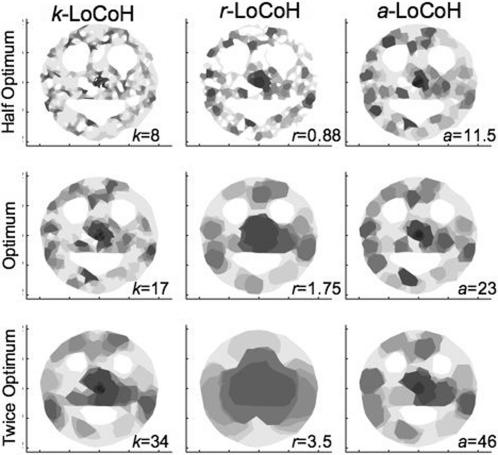
Illustrations of UDs constructed for data set B using *k*-LoCoH, *r*-LoCoH, and *a*-LoCoH methods with half, actual, and twice the optimal *k*, *r* and *a* parameter values. The darkest to lightest areas represent ascending decile areas from the 10^th^ to 100^th^ percentile isopleths.

For the sake of completeness and to permit visual comparisons, the fixed kernel least-square cross validation UDs (95^th^ percentile) are plotted for data sets A, B, and C in Fig. 1, where we see that for all three sets of data, unlike the LoCoH method, the method fails to identify any of the holes.

### Buffalo data

**Figure 8:**
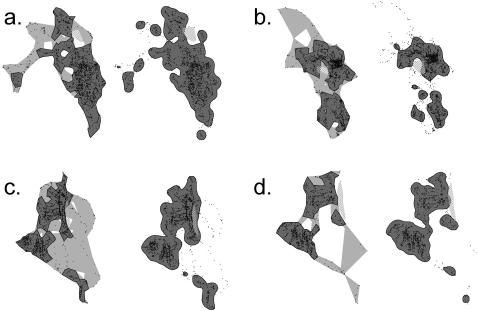
Comparisons of UD constructions using an *a*-LoCoH estimators where the value of the parameter is *â* obtained using the MSHC method (see text for details), and a parametric kernel, where the smoothing parameter *h* is calculated using the ad-hoc method of Silverman (1986). Panels: a. collar T07 and b. collar T15, both in the Satara Region; and c. collar T13 and d. collar T16. both in the Lower Sabie Region. Black circles are GPS collar locations and the hatched shape is the exclosure in a. and b. and the ridge area in c. and d. The left figure of each panel shows the 100% isopleth in light grey and the 95% isopleth in dark grey, using the *a*-LoCoH method. The right figure of each panel shows the 100% kernel in light grey and the 95% parametric kernel in dark grey.

Silverman's parametric kernel method [14] yielded considerably larger area estimates in three of the four cases than the MSHC *a*-LoCoH method (Table 3; Fig. 8a, T07: 244 vs. 173; Fig. 8c, T13: 142 vs. 95; Fig. 8d, T16: 84 vs. 55). Only in one case was the situation reversed (Table 3; Fig. 8b, T15: 121 vs. 153): this appears to be a function of the distribution of the data into a few high-density areas with a few oddly shaped sparse regions. Both the kernel method and the *a*-LoCoH method at the 95% isopleth exclude a number of these points, but the *a*-LoCoH method locally accommodates the denser areas, which, in this case, includes them. The kernel method, applying a constant function, drops all but the 95% densest areas according to a single metric.

**Table 3:** Comparison of the areas in km^2^ estimated for the four buffalo GPS collar sets of data (*n* points) by the 95% and 100% isopleths for nonparametric (LoCoH) and parametric kernel^a^ methods.

Collar	*n*	*HR*	*k-*LoCoH	*r-*LoCoH	*a-*LoCoH		Kernel
parameter			(*k*_1_)	(*r*_1_)	(*a*_1_)	(*â*)	
T07	27	95%	190	268	166	173	244
	89	100%	289 (53)	321 (3475)	236 (32383)	253 (44850)	
T13	25	95%	96	114	89	95	142
	72	100%	238 (51)	144 (1470)	211 (28156)	224 (35000)	
T15	28	95%	126	146	128	153	121
	46	100%	276 (53)	156 (1555)	205 (35684)	257 (72000)	
T16	16	95%	56	46	55	55	84
	75	100%	118 (41)	49 (678)	90 (23401)	90 (23401)	

For the *k*, *r*, and *a*-LoCoH methods the parameters used, as described in the text, are the heuristic values *k*_1_, *r*_1_, and *a*_1_ and the MSHC value *â* (given in parentheses).

a Implemented in Animal Movement Extension for ArcView 3.x [29] using Silverman's ad-hoc method for selecting the smoothing parameter *h* [14].

In the Satara area (Figs. 8a and b) the hashed object embedded in the UD is a large animal exclosure. In the Lower Sabie area, a ridge area that is too steep for buffalo is shown as a hashed object (Figs. 8c and d). Both the MSHC *a*-LoCoH and parametric kernel methods left at least half of the enclosure at Satara uncovered when the 95% isopleth was used as a boundary, but impressively so did the 100% isopleth boundary of the MSHC *a*-LoCoH. (Figs. 8a–b) (the 100% isopleth of the parametric kernel method covers the entire exclosure). The parametric kernel method failed to identify the ridge area embedded within the T13 data in Lower Sabie by completely covering the ridge, while the MSCH *a*-LoCoH 95% isopleth defined the left boundary of the ridge rather clearly and even left the ridge partially uncovered in the 100% isopleth construction (Figs. 8c and d). The MSHC *a*-LoCoH also covered less of the ridge in both the 95% and 100% isopleth constructions than the parametric kernel method did for its 95% isopleth construction.

Note that the symmetric bivariate Gaussian kernel UDs have slightly jagged boundaries because they are generated from an underlying grid, while LoCoH UDs are generated directly from the polygonal elements.

## Discussion

In statistics, nonparametric methods always require fewer assumptions than the corresponding parametric methods. In the case of UD constructions, both parametric and LoCoH kernel methods require common assumptions about data to avoid misinterpretations that come from bias with respect to the way the data are collected. By definition, however, parametric kernel methods always involve additional assumptions about the form of the distributions governing the data that nonparametric methods do not make. Thus, although traditional kernel methods can produce UDs and HRs that follow highly irregular data, they are still based upon parametric kernels that require the investigator to specify their functional form. LoCoH kernels, on the other hand, take their form directly from the data, thereby relieving the investigator of the burden and bias associated with choosing a functional form for the kernels. Further, parametric kernel UD constructions are almost always based on non-compact (i.e. unbounded) symmetric bivariate Gaussian kernels. This implies an ad-hoc decision must be made on which isopleth to use in HR constructions. Although, typically, the 95^th^ percentile is used a 90^th^ percentile boundary may decrease sample size bias [21] and handle poor convergence of area estimates with increasing sample size better. In the latter case, areas of the true home range are invariably omitted. Also, in some cases (as we mention in our methods section) the LSCV method for selecting the best value for the symmetric bivariate Gaussian smoothing or width parameter *h* does not converge and an ad-hoc method must be used to select its value.

Even bounded parametric kernel methods (e.g. Epanechnichov kernels) will always overshoot the data by an amount equal to the value of the kernel radius parameter *h*, no matter how dense the data. On the other hand, LoCoH methods do not overshoot the data, since they use the data directly; and hence converge on true boundaries as the density of data increases [23]. The only errors that LoCoH makes are small: it locally approximates the actual boundary by fitting a line between the two points closest to the true boundary element in question. In essence, our analysis suggests that we should move beyond the assumption, implicit in parametric kernel methods, that all points are internal and recognize that many animals not only visit the boundaries of their range, but may even patrol them as a way of warding of competitors [30].

In a previous publication, we demonstrated the superiority of *k*-LoCoH over symmetric bivariate Gaussian kernel methods [23], whether fixed or adaptive and using Silvermen's ad-hoc or the least-squares-cross-validation algorithm [14], [18]) for selecting the smoothing parameter, for identifying holes in UDs and estimating the areas of HRs. From the results presented here, it is clear that *a*-LoCoH is superior to both *r*-LoCoH and *k*-LoCoH. *A priori* it was not clear to us whether *k* or *r*-LoCoH would be the superior method, but with hindsight, *r*-LoCoH is generally the worst performer because it is essentially a non-parametric kernel method in which all elements are approximately the same size (determined by the value of *r*). On the other hand, the *k*-LoCoH method adapts the size of the kernel elements resulting in smaller kernels in regions with a higher density of locations. The *a*-LoCoH method also has this latter adaptive property; but additionally results in the construction of more robust UDs, because it is the method that is the most insensitive to suboptimal value choices for its kernel parameter (as illustrated in Figs. 5–7). Further, for the datasets we analyzed, our heuristic rule for selecting *a*_1_ typically provided a value that was within 30% of the value a* while our heuristic rules for *r*_1_ and *k*_1_ fluctuated from almost the same to twice as large as the corresponding MSHC values in the case of *k*-LoCoH, and from 1/3 to 3 times less than corresponding MSHC values in the case of *r*-LoCoH. Thus, researchers should feel more confident using *a*_1_ than *r*_1_ or *k*_1_ when *a priori* information on holes is unavailable. Further this confidence in *a*_1_ over *r*_1_ or *k*_1_ still applies even if we modify our heuristic rules for selecting *r*_1_ and *k*_1_ to:

- *r*_1_ is the maximum of all the nearest neighbor distances associated with the data

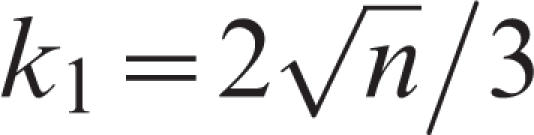
 where *n* is the number of points in the set.

In this modified case, both *r*_1_ and *k*_1_ would only be with 50% of r* and k* respectively. Further, it is not clear that these two rules would remain robust as sample size increase, while, from Table 2, our heuristic rule for *a* seems much less affected by changes to sample size than is the case for *r* and *k*. Thus our overall conclusion is that *a*-LoCoH is the best method unless some compelling reason exists to have all the kernels constructed either from the same number of points (*k*-LoCoH) or for all to be of similar size (*r*-LoCoH).

There has been some confusion about the need for points to have a certain temporal properties. This issue has recently been clarified by Börger et al. [21], and it is becoming clear that important biological information is contained in spatiotemporal autocorrelations of data points [6], [31], [32]. It is important to note, however, that an assumption necessary to ensure the construction of adequate unbiased UDs is that the data points have been collected suitably often to obtain a representative sample of points over time to cover all modes of behavior. If this is not the case, then we have to be careful how we interpret the resulting UDs. In particular, as the sampling intensity decreases, say to twice or four times a day, it becomes increasingly likely that sparse, but regular sampling may coincide with particular activities (e.g. sleeping, drinking, eating) and result in UDs biased towards these activities. Moreover, the scale at which the utilization can be interpreted will still depend on the frequency of data points, even for extremely regularly spaced points. For example, our buffalo data, collected at hourly was still too sparse relative to rate of movement of individuals with regard to identifying small physical obstacles on the landscape, including a small hill that we know is not utilized by buffalo in the Kruger National Park.

As with any numerical method that draws directly upon data, LoCoH HR estimates and UD constructions are only as good as the data they rely upon to carry out the numerical computations. If these data are particularly noisy, then holes will be filled and sharp boundaries blurred. Fortunately, the resolution of GPS data is sufficient to accurately assess the location of sharp boundaries to within a couple of meters when information is collected at appropriately high frequencies (i.e. as they relate to the rate at which individuals move along the boundaries of their range). Assuming high quality data, the great advantage of LoCoH over parametric kernel methods is that LoCoH estimates convergence to true values with increasing sample size. This allows one to study the convergence properties by comparing estimates using a tenth, quarter, half, and all the data. If half the data, for example gives an estimate, within a desired tolerance of the estimate obtained by all the data (e.g. 1% or 0.1%), then one can be confident about the precision of the estimate. Of course, one can also carry out bootstrapping procedures to obtain standard errors [33]. In the end, if one is interested in detecting features in the environment that may influence the way animals utilize space, there is not substitute for using several different methods—both parametric and nonparametric—to construct maps overlaid on geographical and physical feature maps and visually comparing and inspecting the results to identify features that may be attracting, repealing, or excluding individuals.

In summary, LoCoH methods are superior to bounded and, especially, unbounded parametric kernel methods for constructing UDs and HRs because they directly draw upon the actual spatial structure of data that may well be influenced by hard boundaries and irregular exclusionary areas in the environment. Also, our analysis indicates that the *a*-LoCoH nonparametric kernel method is generally superior to the other methods that we considered, both in constructing UDs and in estimating the size of HRs.
